# Impaired aldehyde detoxification exacerbates motor deficits in an alpha‐synuclein mouse model of Parkinson's disease

**DOI:** 10.1002/brb3.3150

**Published:** 2023-07-14

**Authors:** Paul Anthony Martinez, Vanessa Elia Martinez, Sheela Rani, Meredith Murrell, Martin Javors, Jonathan Gelfond, Jonathan Alan Doorn, Elizabeth Fernandez, Randy Strong

**Affiliations:** ^1^ Department of Pharmacology University of Texas Health Science Center San Antonio Texas USA; ^2^ Barshop Institute for Longevity and Aging Studies University of Texas Health Science Center San Antonio Texas USA; ^3^ Center for Biomedical Neuroscience University of Texas Health Science Center San Antonio Texas USA; ^4^ Department of Psychiatry University of Texas Health Science Center at San Antonio San Antonio Texas USA; ^5^ Department of Medicine University of Texas Health Science Center San Antonio Texas USA; ^6^ Department of Epidemiology & Biostatistics University of Texas Health Science Center San Antonio Texas USA; ^7^ Geriatric Research, Education, and Clinical Center South Texas Veterans Health. Care Network San Antonio Texas USA; ^8^ Department of Pharmaceutical Sciences and Experimental Therapeutics, College of Pharmacy The University of Iowa Iowa City Iowa USA

**Keywords:** aldehyde dehydrogenase, aldh1a1, aldh2, alpha‐synuclein, DOPAL, oligomers

## Abstract

**Introduction:**

The discovery of biogenic aldehydes in the postmortem parkinsonian brain and the ability of these aldehydes to modify and cross‐link proteins has called attention to their possible role in Parkinson's disease. For example, many in vitro studies have found that the aldehyde metabolite of dopamine, 3,4‐dihydroxyphenylacetaldehyde (DOPAL), induces the formation of stable, neurotoxic alpha‐synuclein oligomers.

**Methods:**

To study this in vivo, mice deficient in the two aldehyde dehydrogenase enzymes (Aldh1a1 and Aldh2, DKO) primarily responsible for detoxification of DOPAL in the nigrostriatal pathway were crossed with mice that overexpress human wild‐type alpha‐synuclein. DKO overexpressing human wild‐type alpha‐synuclein (DKO/ASO) offspring were evaluated for impairment on motor tasks associated with Parkinsonism.

**Results:**

DKO/ASO mice developed severe motor deficits greater than that of mice overexpressing human wild‐type alpha‐synuclein alone.

**Conclusion:**

These results provide evidence to support the idea that biogenic aldehydes such as DOPAL interact with human wild‐type alpha‐synuclein, directly or indirectly, in vivo to exacerbate locomotor deficits in Parkinson's disease.

## Graphical abstract

The biogenic aldehyde metabolite of dopamine, DOPAL, exacerbates motor impairment in mice overexpressing human wild‐type alpha‐synuclein. Molecular assays from this study indicated reduced expression of tyrosine hydroxylase and increased expression of the neuroinflammatory marker, glial fibrillary acidic protein, in the same group of mice. Our findings suggest that DOPAL interacts with alpha‐synuclein either directly or indirectly to exacerbate motor impairment.

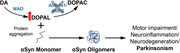

## 1 INTRODUCTION

Parkinson's disease (PD) is the second most common neurodegenerative disease after Alzheimer's disease (AD). PD is characterized by the progressive loss of nigrostriatal dopamine (DA) neurons which is accompanied by deficits in locomotor function. The cardinal motor symptoms, bradykinesia, resting tremor, rigidity, and postural instability, appear after ∼70%–80% of DAergic neurons are lost (Bernheimer et al., 1973; Dauer & Przedborski, 2003; Greenamyre et al., 2001; Marsden, 1992). The presence of Lewy bodies (LBs), intraneuronal protein deposits in surviving DA neurons, is a pathological hallmark of PD. Over 90 proteins have been identified within LBs, with the presynaptic protein alpha‐synuclein (αSyn) identified as a primary constituent (Wakabayashi et al., 2013).

The cause of PD remains unclear. Systemic deficiency of mitochondrial complex I (NADH‐dehydrogenase) (Keeney et al., 2006; Kotraiah et al., 2013; Mizuno et al., 1989; Schapira et al., 1989) and increased levels of oxidative damage in the PD brain postmortem suggest a role of oxidative stress in PD (Alam, Daniel, et al., 1997; Alam, Jenner, et al., 1997; Castellani et al., 2002; Yoritaka et al., 1996). Free radicals and biogenic aldehydes, such as 3,4‐dihydroxyphenylacetaldehyde (DOPAL), are known to act as signaling molecules under physiological conditions and are associated with oxidative damage under pathological conditions. Unlike short‐lived free radicals, long‐lived biogenic aldehydes (hereinafter referred to as aldehydes) can act as second messengers of reactive oxygen species (Jaganjac et al., 2013). These highly reactive electrophilic species are toxic to cellular components resulting in irreversible damage. Importantly, aldehydes have the capacity to act at the site of formation or on distant targets by crossing cell membranes (Esterbauer et al., 1991). Altered ratios of DA metabolites found in the nigrostriatal pathway of patients afflicted with PD and diffuse LB disease implicate decreased DOPAL detoxification and suggest elevated levels of this metabolite during pathogenesis (Burke et al., 2004; Castellani et al., 2002; Goldstein et al., 2011; Yoritaka et al., 1996).

The aldehyde intermediate of dopamine metabolism catalyzed by monoamine oxidase, DOPAL, is hypothesized to accumulate in the brain of individuals who died with PD (Castellani et al., 2002; Goldstein et al., 2011). DOPAL is capable of modifying proteins, potentially leading to neurotoxicity (Anderson et al., 2016; Burke et al., 2008; Doorn & Petersen, 2002; Jinsmaa et al., 2011; Malecki et al., 2000; Mexas et al., 2011). Aldehyde accumulation is in part attributable to insufficient aldehyde detoxification by NAD^+^‐dependent aldehyde dehydrogenase (ALDH in humans) enzymes in the nigrostriatal pathway, potentially due to a combination of oxidative stress and/or genetic and environmental factors (Fitzmaurice et al., 2014; Florang et al., 2007; Galter et al., 2003; Jinsmaa et al., 2009; Kotraiah et al., 2013). The neurodegenerative effects of biogenic aldehydes in vivo is supported by previous studies in *Aldh1a1^−/−^ X Aldh2^−/−^* (DKO) mice (Wey et al., 2012). Aldh deficiency in DKO mice resulted in locomotor dysfunction that was responsive to levodopa treatment. These locomotor deficits were accompanied by the loss of tyrosine hydroxylase (TH)‐immunoreactive neurons and were preceded by elevated levels of DOPAL. The downstream mechanisms by which these aldehydes contribute to the loss of DAergic neurons and motor impairment in this animal model are unclear.

The presence of αSyn in LBs and its propensity to form aggregates under pathological conditions implicate αSyn in PD. Although αSyn's function remains unclear, it is well accepted that αSyn plays a role in neurotransmitter release (Abeliovich et al., 2000; Nemani et al., 2010; Perez et al., 2002; Yavich et al., 2006, 2005, 2004). The formation of αSyn protein aggregates may not only alter αSyn's function, but trigger neurotoxicity. In vitro studies provide evidence that DOPAL is capable of modifying and cross‐linking proteins, including αSyn (Burke et al., 2008; Doorn & Petersen, 2002; Anderson et al., 2011; Jinsmaa et al., 2011; Nasstrom et al., 2011; Qin et al., 2007; Rees et al., 2009; Werner‐Allen et al., 2016). The small 140 amino acid chain that makes up αSyn includes histidine and lysine residues that are reactive with biogenic aldehydes such as DOPAL (Doorn & Petersen, 2002; Follmer et al., 2015; Rees et al., 2009; Werner‐Allen et al., 2016). Additionally, in vitro studies support the idea that DOPAL is capable of inducing the formation of toxic αSyn oligomers that inhibit the subsequent formation of fibrils, a conformation believed to act as a neuroprotective mechanism (Burke et al., 2008; Follmer et al., 2015; Karpinar et al., 2009; Qin et al., 2007; Winner et al., 2011). The mechanism of neurotoxicity in vivo is unknown. In the present study, we tested the hypothesis that elevated levels of DOPAL exacerbate motor impairment in mice that overexpress human wild‐type (WT) αSyn (ASO). To test this hypothesis, we created aldehyde‐deficient mice overexpressing human WT alpha‐synuclein in which we cross‐bred ASO mice, with DKO mice that have enhanced levels of DOPAL.

## 2 MATERIALS AND METHODS

### 2.1 Animals and ethics statement

All animals were maintained, and experiments were conducted, in accordance with the Institutional Animal Care and Use Committee at the University of Texas Health Science Center, the South Texas Veterans Health Care System (San Antonio, TX), and the 1996 Guide for the Care and Use of Laboratory Animals of the National Institutes of Health. Male mice were multi‐housed (up to five per cage) in a ventilated caging system with paper bedding. Animals were maintained on a 12‐h light‐dark cycle at 23–25°C, and food and water were provided ad libitum throughout the study. Aldh1a1 mice developed by Duester (2001) were crossed with Aldh2 mice previously generated in our lab (Fernandez et al., 2006) to develop DKO mice as described in Wey et al. (2012). DKO overexpressing human WT alpha‐synuclein (DKO/ASO) mice were generated by first crossing DKO mice with Thy1‐αSyn mice (Line 61) generated by Eliezer Masliah (Rockenstein et al., 2002) to obtain Aldh1a1^+/−^ x Aldh2^+/−^ and Thy1‐αSyn^Tg/NTg^ mice. DKO mice were generated on a C57Bl6 background. The Thy1‐αSyn model was generated on a C57Bl6/DBA2 background and fully backcrossed onto a C57Bl6 background (Chesselet et al., 2012). Cross‐breeding of Aldh1a1^+/−^ x Aldh2^+/−^ and Thy1‐αSyn^Tg/NTg^ mice generated Aldh1a1^+/+^ x Aldh2^+/+^ x Thy1‐αSyn^NTg/NTg^ (WT), Aldh1a1^−/−^ x Aldh2^−/−^ x Thy1‐αSyn^NTg/NTg^ (DKO), Aldh1a1^+/+^ x Aldh2^+/+^ x Thy1‐αSyn^Tg/NTg^ (alpha‐synuclein overexpressing, ASO), and Aldh1a1^−/−^ x Aldh2^−/−^ x Thy1‐αSyn^Tg/NTg^ (DKO/ASO) mice. Thus, the genetic background of all mice was identical.

### 2.2 Genotyping

Mouse genotypes were confirmed by polymerase chain reaction (PCR) using DNA extracted from 0.5 cm tail snips. Tail snips were lysed in 200 μL of Direct PCR lysis reagent (102T, Viagen Biotech Inc.) containing 1.0 mg/mL proteinase K (502‐PK, Viagen Biotech Inc.). Tail snips were then incubated overnight at 60°C. The reaction was stopped by incubating at 85° for 1 h. Samples were clarified at 9000 RPM. Lysate supernatant containing genomic DNA was used for Aldh1a1, Aldh2, and Thy1 (or ASO) PCR reactions. Primers in the presence of GoTaq Green Master Mix were amplified by an adapted touchdown PCR protocol using the following conditions: Stage 1, denaturation for 50 s at 95°C, annealing for 30 s beginning at 71°C, extension for 60 s at 72°C. The annealing stage was set for 16 cycles beginning at 71°C and decreasing by 1°C increments. Stage 2, denaturation for 50 s at 95°C, annealing for 50 s at 55°C, extension for 50 s at 72°C. The second annealing stage was set for 19 cycles fixed at 72°C. A hold of 5 min at 72°C between Stages 1 and 2 was set. Amplified products were visualized on a 1.2% agarose gel.

### 2.3 Behavioral and physiological testing

Four‐month‐old (*n* = 14–15 per group) and 6‐month‐old (*n* = 6–7 per group) male mice from each genotype were used for the study. The location of the Thy1‐αSyn transgene in the X chromosome, and the possibility of random inactivation of the gene, may lead to attenuated motor impairment in female mice. For this reason, we have chosen to use only male mice (Chesselet et al., 2012). Four‐month‐old mice were assessed for frailty and tested using, pole test, gait analysis, grip strength, and accelerated rotarod.

Four‐month training and testing was carried out over the course of 3 weeks. On days 1 and 2, frailty assessment was carried out. Animals were given at least 24 h prior to beginning the 24‐h activity that was completed between days 2 and 5. Pole test training was conducted on days 8 and 9 followed by pole testing on day 15. Rotarod training was also conducted during the same week following pole test training and testing. Animals were given 1 h between pole task and rotarod training. Gait analysis and grip strength were conducted on day 17 with 1 h rest between tests. On day 18, body weights were collected and rotarod testing was conducted. For each behavioral task, the total time required to test 60 animals was determined. This time was divided to include morning testing hours. Each set of animals tested for the day for each test included approximately equal number of animals from WT, DKO, ASO, and DKO/ASO groups.

A separate group of 6‐month‐old mice was tested for sensorimotor function using the adhesive removal test. All tests were conducted in the light cycle on separate days with the exception for gait and grip strength tests. These tests were conducted on the same day, with grip strength tested 30–60 min after completion of gait testing.

#### 2.3.1 31‐ITEM frailty assessment

One in three PD patients display frailty during aging suggesting that frailty is not absolute in PD patients. These numbers, however, represent nearly fivefold greater occurrence of frailty than the aged population (Ahmed et al., 2008). Whitehead et al. (2014) have described frailty as “a state of increased vulnerability to adverse health outcomes for people of the same age.” Frailty is evaluated using several parameters to calculate a frailty index both in the clinic and mice (Whitehead et al., 2014). Frailty in mice was assessed using a 31‐item frailty assessment developed in the laboratory of Susan Howlett (Whitehead et al., 2014). Parameters were used to assess and score the animals’ general body condition, ability to carry out simple motor tasks, and responses to sensory stimuli. A score of “0” was recorded where no deficit was observed, “0.5” for a mild deficit and “1” for a severe deficit. A frailty index score was determined by dividing the sum of parameter scores by the total number of parameters.

#### 2.3.2 Accelerating rotarod

Performance on the accelerating rotarod was used as a measure of balance and motor coordination. Mice were trained to run on a Rotamex‐5 rotarod (Columbus Instruments) equipped with a mouse spindle (3.0 cm × 9.5 cm). The rotarod was programmed to accelerate from 4 to 40 revolutions per minute over the course of 300 s. Mice were trained for 5 days, rested for 2 days, and tested on the eighth day. Each training and testing session consisted of three trials. The average latency to fall (seconds) was calculated based on three trials per day.

#### 2.3.3 Pole test

The pole test, which is a measure of bradykinesia, has been considered one of most sensitive behavioral tasks in the detection of motor disturbances resulting from nigrostriatal dysfunction (Meredith & Kang, 2006; Ogawa et al., 1985). The vertical pole test carried out in this study follows the general protocol outlined by Fleming et al. (2004). For this task, animals were placed near the top of a pole 50 cm long with the nose facing upward. Animals were trained for 2 days to turn and descend the length of the pole down to the base and tested on the third day. Training and testing consisted of five trials per day with a maximum time of 3 min per trial. Video files, recorded on the test day, were analyzed using the SMART video tracking software (Panlab|Harvard Apparatus) for events (turn, fall, and slide), and latency to complete the task. The resulting data are commonly expressed by plotting the latency to turn and the latency to complete the task. Despite following previous training protocols outlined by Fleming et al. (2004), the tendency for mice overexpressing αSyn to fall and inconsistent turns between individual mouse trials necessitated modifying the conventional analysis. To maintain the major component of the pole test important in assessing bradykinesia, we plotted the latency to turn based on successful turns. In addition, the turn and fall percentage of each genotype was plotted to fully portray any resulting deficits.

#### 2.3.4 Adhesive removal test

Orofacial and forelimb movement required for the adhesive removal test has been linked to dopamine‐dependent behavior observed in nest building in MPTP mouse models of PD (Sager et al., 2010; Sedelis et al., 2000). Therefore, we included the adhesive removal test as a test of sensorimotor function. The adhesive removal test was carried out in 6‐month‐old mice following a modified protocol published by Lu et al. (2009). A circular adhesive label (Thermo Fisher Scientific, 0.375″) was placed on the animal's snout. The time for the animal to contact the label (contact time) and the time to remove the label (removal time) were recorded. The latency to remove the label was calculated by subtracting the contact time from the removal time. Each animal received three trials with a maximum completion time of 60 s. Two minutes were allowed between trials.

#### 2.3.5 Automated gait analysis

The use of gait analysis in animals is translatable to gait measured in clinical research (Brooks & Dunnett, 2009). Here, we utilized automated treadmill gait analysis using the TreadScan system (CleverSys Inc.) to detect gait anomalies in mice. Mice were placed on a transparent belt moving at 9 cm/s for 10 s. Video software recorded each test subject, and the TreadScan gait analysis software was used to analyze each animal's stride length and track width in millimeters. The stride length is calculated as the distance traveled of either the forelimbs or hindlimbs. The track width is the distance between the trajectory of each step from either the forelimbs or hindlimbs.

#### 2.3.6 24‐Hour locomotor activity

Spontaneous horizontal and vertical activity was assessed for 24 h using the Opto‐M3 activity system (Columbus Instruments). Animals were placed in a home‐like cage environment equipped with infrared beams with access to food and water.

### 2.4 Preparation of brain tissue

Euthanasia of mice was carried out using carbon dioxide inhalation followed by cervical dislocation. Mice were decapitated to harvest select brain regions. Isolated tissues were snap‐frozen in a dry ice‐acetone bath and then stored at −80°C until further analyses.

#### 2.4.1 SDS‐PAGE/western blot

*Aldh1a1, Aldh2, pS129‐αSyn, and TH*: Proteins were extracted from tissue using 1% nonidet‐P40 (Thermo Fisher Scientific) lysis buffer at 10% w/v containing protease/phosphatase inhibitors (Calbiochem Set III and V). Protein content was determined using the micro‐BCA protein assay kit (Thermo Fisher Scientific). Samples were prepared in lithium dodecyl sulfate sample buffer (NP0008, Invitrogen) and β‐mercaptoethanol reducing agent (Sigma‐Aldrich). Approximately 30–40 μg of protein was separated on a 4%–12% Bis‐Tris SDS‐PAGE gel (Invitrogen). Electrophoresis was run in MOPS buffer with antioxidant (NP0005, Invitrogen) at 175 volts for 60 min followed by a cold transfer onto Immobiolon‐FL polyvinylidene fluoride membrane (IPFL00010, EMD Millipore) at 100 volts for 120 min. Assays that utilized total protein normalization were determined using the REVERT total protein stain kit (926–11010, LI‐COR Biosciences) prior to proceeding to the blocking step. Membranes were blocked with Odyssey PBS blocking buffer (927–40000, LI‐COR Biosciences) and then probed with Aldh1a1 (GTX123973, GeneTex), Aldh2 (ab108306, Abcam), recombinant anti‐αSyn (phospho‐S129; ab51253, Abcam), tyrosine hydroxylase (T2928, Sigma‐Aldrich), β‐actin (CST‐3700, Cell Signaling Technology), or GAPDH (NB300‐323, Novus Biologicals, LLC) primary antibodies overnight at 4°C. The following day, the membrane was washed and probed with the appropriate fluorescent secondary antibody (IR680 or IR800, LI‐COR Biosciences) for 60 min at room temperature. Fluorescent detection of proteins was visualized using the Odyssey Imaging System (LI‐COR Biosciences) and analyzed using Image Studio Lite software (Li‐COR Biosciences).

*Glial fibrillary acidic protein* (*GFAP)*: 10 μg protein was used for immunoblotting of GFAP and β‐actin (ACTB). GFAP protein was detected using primary mouse GFAP monoclonal antibody (SC‐33673, Santa Cruz Biotechnology, Inc.), followed by the secondary antibody, goat anti‐mouse IgG‐IRDye 800CW (926–32210, LI‐COR Biosciences). The same blot was reprobed for the housekeeping protein β‐actin using a primary rabbit monoclonal Ab (CST‐4970, Cell Signaling Technology) followed by the secondary antibody, donkey anti‐rabbit IgG‐IRDye‐680RD (926–68073, LI‐COR Biosciences). Images obtained on Li‐Cor Odyssey CLx system showing a 50 kDa GFAP and a 42 kDa ACTB bands were quantified using the Image Studio software (LI‐COR Biosciences). Results are expressed as ratio of GFAP to ACTB and plotted as percent of WT control.

#### 2.4.2 Quantitative PCR

Measurement of glial fibrillary acidic protein (GFAP) mRNA levels were measured in the striatum by quantitative PCR (qPCR) as a measure of GFAP gene expression. Total RNA was isolated using the Quick RNA Miniprep kit (R1054, Zymo Research) as described by the manufacturer's protocol. Total RNA was treated with RNase‐free DNase I, followed by DNase removal using RNA Clean & Concentrator kit (R1013, Zymo Research). Total RNA was quantified on a Nanodrop, and 500 ng RNA was converted to cDNA using the SensiFast cDNA Synthesis kit (BIO‐65053, Bioline). Reaction controls included RNA template only (no reverse transcriptase) and reverse transcriptase only (no RNA template) in 20 μL volume. Following a 15‐min incubation period at 42°C, cDNA quality was examined. One μL of the reaction mix containing cDNA was used as template for PCR with GAPDH primers and the DreamTaq Green Hotstart PCR master mix (K9021, Thermo Fisher Scientific) and analyzed on 2% agarose E‐Gel (A42135, Thermo Fisher Scientific). No bands were detected either in negative control (no template) or in the samples without the reverse transcriptase enzyme, but a 200 base pair band was seen in all RT reactions with cDNA‐containing samples.

#### 2.4.3 High‐performance liquid chromatography with electrochemical detection

*Chemicals and Reagents*. High‐performance liquid chromatography‐grade acetonitrile, methanol, and o‐phosphoric acid were purchased from Fisher Scientific (Fair Lawn). Water purified with a Milli‐Q Water System was used in the assay (Millipore). Ethylenediamine tetra acetic acid (EDTA) and octanesulfonic acid (OSA) were obtained from Sigma‐Aldrich Corp. DOPAL was purchased from Santa Cruz Biotech (SC‐391117, Santa Cruz Biotechnology Inc.). The internal standard, DHBA, was obtained from Sigma‐Aldrich Corp.

*HPLC System*. The high performance liquid chromatography with electrochemical detection (HPLC‐EC) system consisted of an ESA model 584 HPLC pump, ESA model 542 autosampler, and an ESA Coulochem III detector with a 5014B microdialysis cell. The detector was set to a voltage of 220 mV and an *R* value of 1 μA. The ACE C18 analytical column (3.0 × 75 mm, 3μ) was purchased from Mac Mod Analytical (Chadds Ford). The mobile phase contained 92% water, 7.5% acetonitrile, 0.5% o‐phosphoric acid, 0.05% 50 mM EDTA, 0.05 g OSA (per liter) and was at a pH of 2.5. The flow rate of the mobile phase was set at 0.4 mL/min. The metabolites eluted from the column in the following order: DHBA at 1.9 min and DOPAL at 2.9 min.

*Quantification of DOPAL in brain tissue*. Brain tissue for samples, calibrators, and controls were weighed into polypropylene tubes, where 10× volume of a 75% methanol solution was added. Samples were homogenized, and 100 μL of each was aliquoted into clean polypropylene tubes. Ten microliters of DHBA (10 μg/mL) for internal standard was then added to each sample. Samples were vortexed for 30 s and then centrifuged at 17,000 g for 5 min in a Fisher Scientific accuSpin Micro 17 centrifuge. Five microliters of the supernatant was injected into the HPLC system.

The peak area ratio of each analyte and the internal standard, DHBA, were determined using the Waters Empower chromatography software. Concentrations in unknown samples were quantified by comparing peak area ratios against the linear regression ratios of calibrators at 0, 5, 10, 25, 50, 100, 500, and 1000 ng/mL. The amount of each analyte was reported as ng/mL and converted to μg/g based on the tissue mass of each sample.

### 2.5 Statistical analysis

The statistical significance of results was analyzed using GraphPad prism 8.0.0 statistical analysis software. Normally distributed data were analyzed using a one‐way analysis of variance followed by a pairwise comparison using the Tukey's or Bonferroni post hoc test. Non‐parametric data were analyzed using the Kruskall–Wallis test followed by a pairwise comparison using the Dunn's multiple comparison post hoc test.

The adhesive removal test was conducted using the generalized estimating equation (GEE). The GEE was fit with Gaussian distribution and with long link function. Models were first used without interactions to test for four types of correlation structures: independence, exchangeable, AR1, and unstructured. Correlation structures were based on quasi‐information criterion goodness of fit statistic. We selected unstructured for the Contact subset and AR1 for the removal and latency to remove subset. The results display the estimated coefficients and robust standard error of the mean (SEM) for the regression parameters. The Wald statistic was used to test whether the parameter is significant. The reference level for trial and group is the first trial and WT group, respectively. These were followed with a pairwise *comparison* using Tukey adjustment. For the GEE analyses, results are reported as estimate ± SEM, *p‐*value. Graphical representation of statistically significant data for all analyses are expressed as: *p ≤ .05; **p ≤ .01; ***p ≤ .001; ****p ≤ .0001.

## 3 RESULTS

### 3.1 Genotype verification by PCR

**FIGURE 1:**
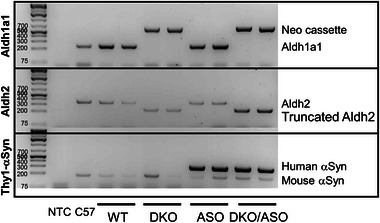
Confirmation of transgenic mouse models. Polymerase chain reaction (PCR) was used to confirm the deletion of Aldh1a1 and Aldh2 and/or the insertion of human WT‐αSyn (hWT‐αSyn) in WT (lanes 3–4), DKO (lanes 5–6), ASO (lanes 7–8), and DKO/ASO (lanes 9–10) mice. A no template control (NTC; lane 1) and C57Bl/6 lysate (C57; lane 2) were used as controls. The DNA agarose gel is a representation of the Aldh1a1 (top panel; 250 bp), Aldh2 (middle panel; 350 bp), and the Thy1‐αSyn (bottom panel; 349 bp). PCR reactions were used to obtain the corresponding DNA transcripts amplified from tail snips.

DNA extracts from mouse tail snips were amplified using the PCR to screen genotypes in the current study. Targeted DNA fragments were separated and visualized by gel electrophoresis. A representation of WT, DKO, ASO, and DKO/ASO genotypes is shown in Figure 1.

WT copies of Aldh1a1 and Aldh2 in WT and ASO mice are visible at 250 and 350 bp, respectively. These bands are absent in extracts from DKO and DKO/ASO mice. In the Aldh1a1 reaction, DKO and DKO/ASO mice showed a 600 bp band corresponding to the neomycin cassette used to disrupt Aldh1a1 expression. Similarly, a non‐functional truncated Aldh2 transcript band was observed at 250 bp for both DKO and DKO/ASO mice. The 349 bp, Thy1‐αSyn insert containing human WT αSyn (hWT‐αSyn), is only visible in extracts from ASO and DKO/ASO mice. All mice had functional copies of endogenous mouse αSyn.

### 3.2 Effect of genotype on protein expression

**FIGURE 2:**
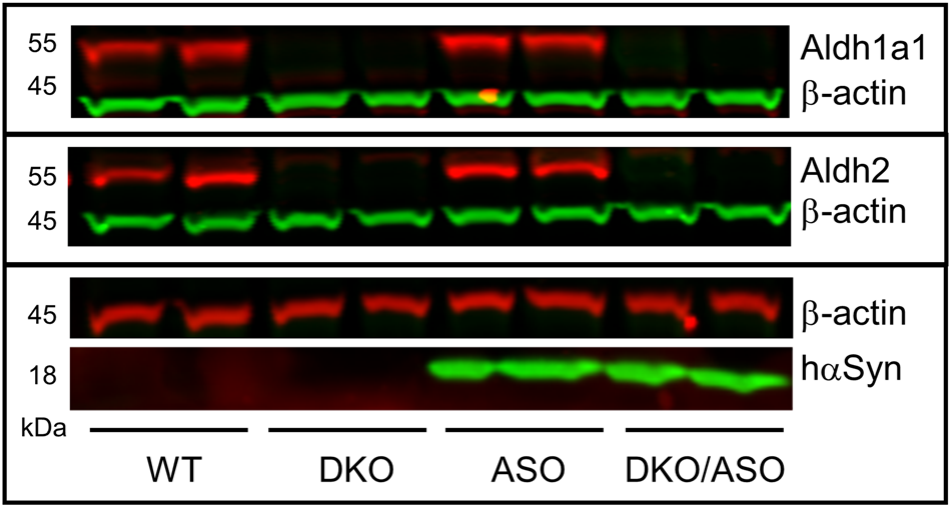
Protein expression of Aldh and hWT‐αSyn in the nigrostriatal pathway. Immunoblot of Aldh1a1, Aldh2, and hWT‐αSyn protein expression in the midbrain of WT, DKO, ASO, and DKO/ASO mice. Aldh1a1 and Aldh2 protein expression is observed at 55 kDa, and hWT‐αSyn driven by the Thy1‐αSyn construct is visualized at 18 kDa. Beta‐actin was used as a loading control.

Midbrain protein expression of Aldh1a1, Aldh2, and hWT‐αSyn from each genotype was determined by western blot as represented in the immunoblot in Figure 2. Aldh1a1 and Aldh2 proteins visible at ∼55 kDa are highly expressed in WT and ASO mice. In contrast, these proteins are absent in DKO and DKO/ASO genotypes. While all genotypes expressed murine αSyn protein, hWT‐αSyn was expressed only in ASO and DKO/ASO mice.

### 3.3 Effect of genotype on DOPAL content

**FIGURE 3:**
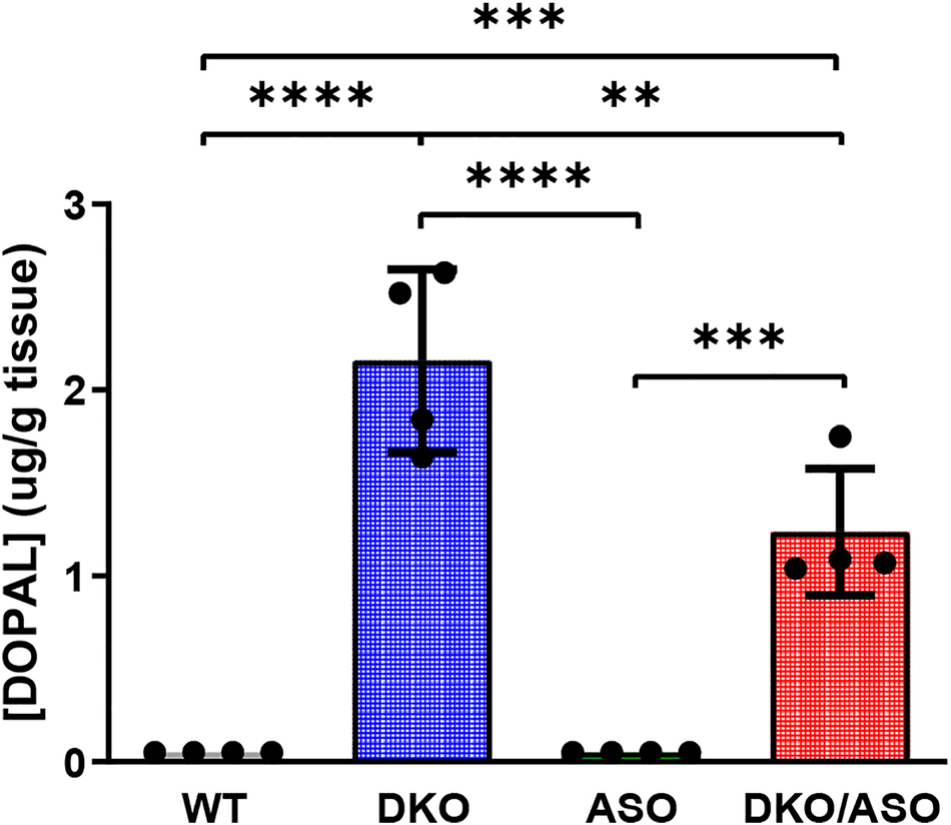
Effect of genotype on DOPAL accumulation in the striatum. The concentration of striatal DOPAL was measured in WT, DKO, ASO, and DKO/ASO mice by HPLC‐EC. Scatter bars represent the average concentration ± SD from *n* = 4 per group. Statistically significant differences are indicated as follows: *p ≤ .05; **p ≤ .01; ***p ≤ .001; ****p ≤ .0001.

The effect of genotype on striatal DOPAL content is shown in Figure 3. DOPAL content was significantly greater (*F*_3,12_ = 46.71, *p* < .0001) in the DKO and DKO/ASO groups compared to the WT (*p* < .0001) and ASO (*p* < .0001) groups.

### 3.4 Effect of genotype on frailty

**FIGURE 4:**
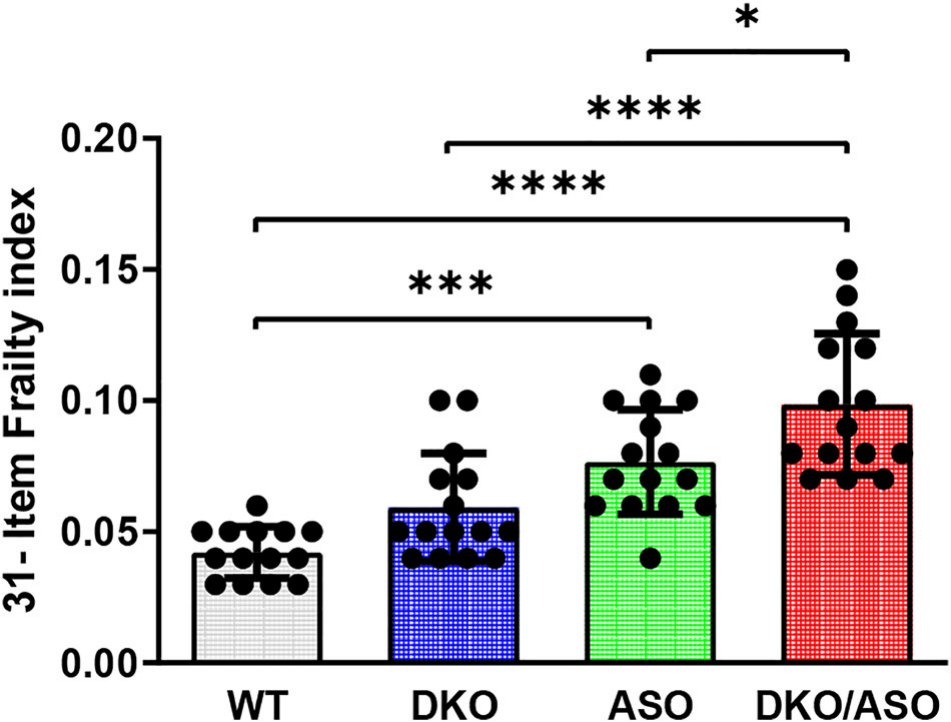
Effect of genotype on frailty. Deficit accumulation based on a 31‐item assessment was used to determine frailty index in mice. Scatter bars represent the mean ± SD of frailty index from *n* = 14–15 mice per group. Statistically significant differences are indicated as follows: *p ≤ .05; ***p ≤ .001; ****p ≤ .0001.

Frailty assessment as measured by the frailty index is shown in Figure 4. There was a significant effect of genotype on frailty (*F*_3,55_ = 20.52, *p* < .0001) in the ASO group in comparison to WT controls (*p* = .0002). Similarly, the DKO/ASO group with a frailty index score of 0.099 had significantly greater frailty scores compared to the WT (0.042) and DKO (0.059) groups (WT, *p* < .0001; DKO, *p* < .0001). We observed a significantly greater frailty score in the DKO/ASO group in comparison to the ASO group (*p =* 0.0230) with a frailty index score of 0.077. There was no significant difference between the DKO and control groups.

### 3.5 Effect of genotype on accelerated rotarod performance

**FIGURE 5:**
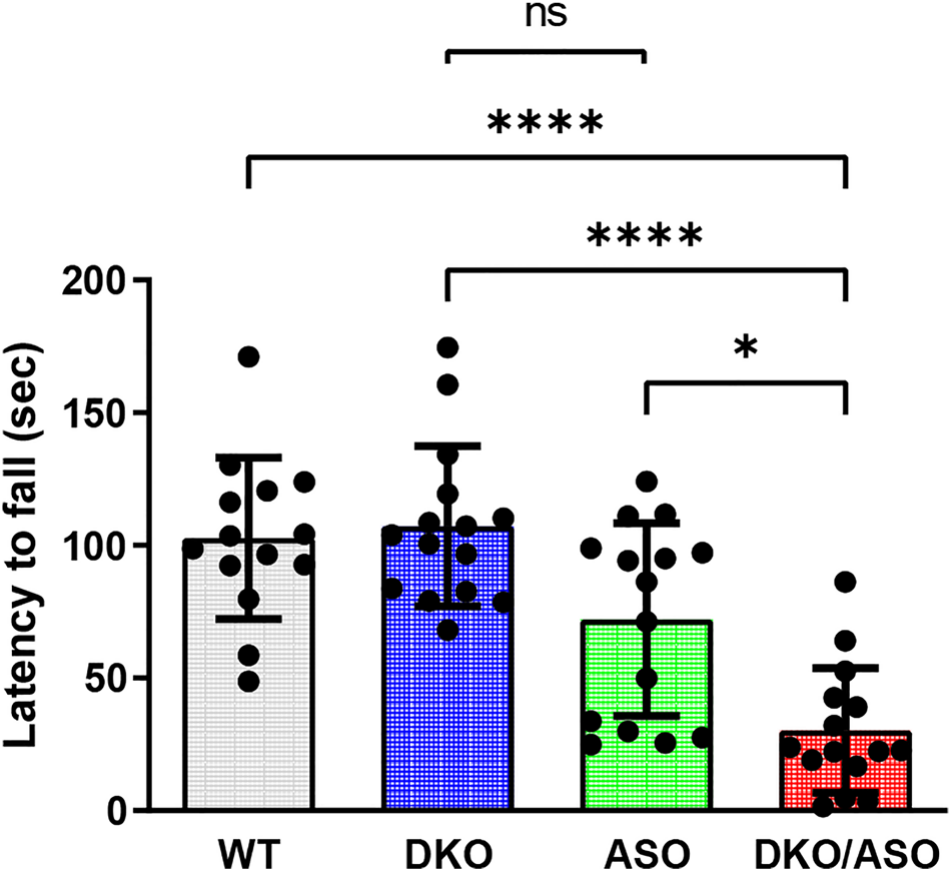
Effect of genotype on motor function assessed by accelerated rotarod. The average latency to fall from three trials was plotted. Scatter bars represent the mean ± SD of the latency to fall from *n* = 14–15 mice per group. Statistically significant differences are indicated as follows: *p ≤ .05; ****p ≤ .0001.

Measures of accelerated rotarod performance are plotted in Figure 5. The DKO/ASO group spent significantly less time on the accelerated rotarod when compared to any of the other groups (WT, *p* < .0001; DKO, *p* < .0001; ASO, *p* = 0.0263). DKO and ASO groups were not significantly different in time spent on the rotarod.

### 3.6 Effect of genotype on the pole test

**FIGURE 6:**
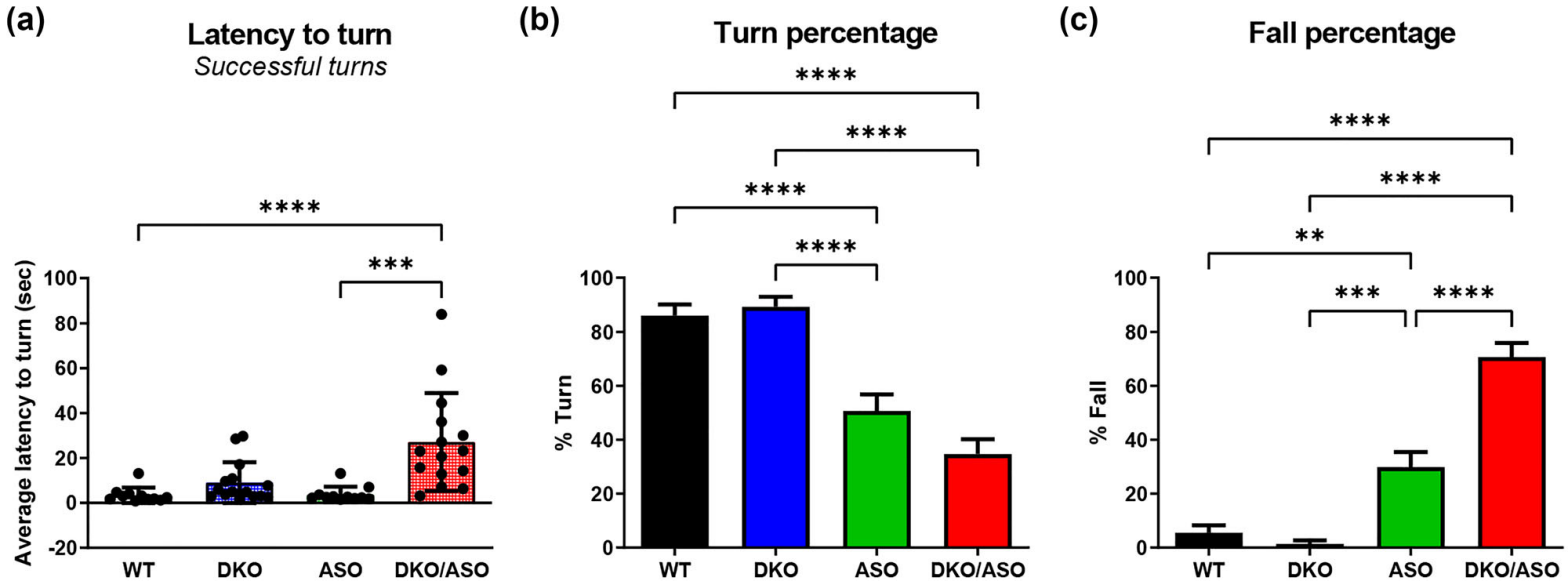
Effect of genotype on motor function assessed by the pole test. (a) The average latency to turn was calculated based on the number of successful turns from five trials. (b) The average turn percentage and (c) average fall percentage per group were also plotted. Scatter bars represent the mean ± SD from *n* = 11–15 mice per group. Statistically significant differences are indicated as follows: **p ≤ .01; ***p ≤ .001; ****p ≤ .0001.

Results for the vertical pole test are shown in Figure 6. As shown in Figure 6a, the DKO/ASO group took significantly longer to turn at the top of the pole in comparison to WT and ASO groups (*p* < .0001 for each). Figure 6b shows that the turn percentage for the ASO and DKO/ASO groups was 51% and 35%, respectively. This resulted in a significantly lower turn percentage in comparison to the WT and DKO groups (*p* < .0001 for each). There was no difference between the ASO and DKO/ASO groups. The fall percentage for each group is plotted in Figure 6c. Both ASO and DKO/ASO mice fell significantly more often than the WT and DKO groups with fall percentages of 30% (WT, *p* = .0077; DKO, *p* = .0008) and 71% (*p* < .0001 for each), respectively.

### 3.7 Effect of genotype on the adhesive removal test

**FIGURE 7:**
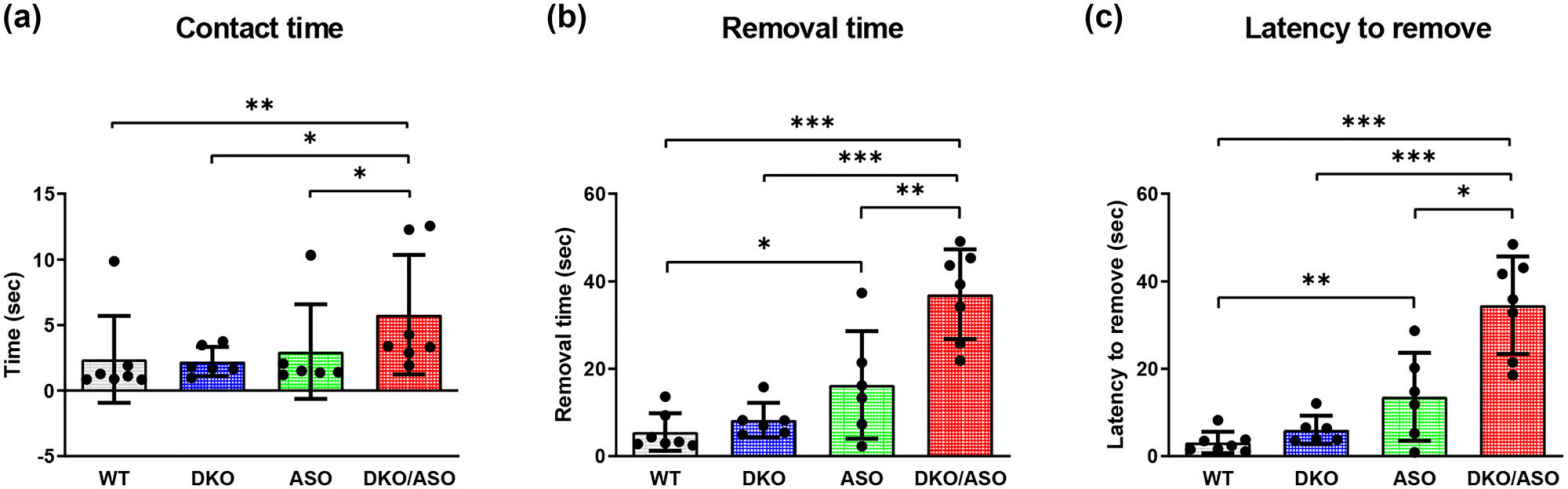
Effect of genotype on the adhesive removal test. The average time for each animal to make (a) contact and (b) remove the adhesive label was used to calculate the (c) average latency to remove the label. Scatter bars represent the mean ± SD from 6‐month‐old mice (*n* = 6–7 per group). Statistically significant differences are indicated as follows: *p ≤ .05; **p ≤ .01; ***p ≤ .001.

The adhesive removal test was used as a measure of sensorimotor function. In comparison to WT controls, the ASO group took significantly longer to complete the adhesive removal task (Figure 7b). This significance persisted after correcting for animal contact time with the adhesive label as shown in Figure 7c (0.7 ± 0.2 s, *p* = .03, 1.5 ± 0.4 s, *p* = .003). The ASO group did not differ in contact time from any other group (Figure 7a). The DKO/ASO group took significantly longer than any other group to make contact with the label (Figure 7a; WT, 1.5 ± 0.4 s, *p* = .002; DKO, 1.1 ± 0.4 s *p* = .02; ASO, 1.2 ± 0.4 s *p* = .04), complete the task (Figure 7b; WT, 1.5 ± 0.2 s, *p* < .001; DKO, 1.2 ± 0.1 s, *p* < .001; ASO, 0.8 ± 0.2 s, *p* = .01), and showed higher latency to remove the label (Figure 7c; WT, 2.3 ± 0.4 s, *p* < .001; DKO, 1.6 ± 0.2 s, *p* < .001; ASO, 0.8 ± 0.3 s, *p* = .01).

### 3.8 Effect of genotype on gait

**FIGURE 8:**
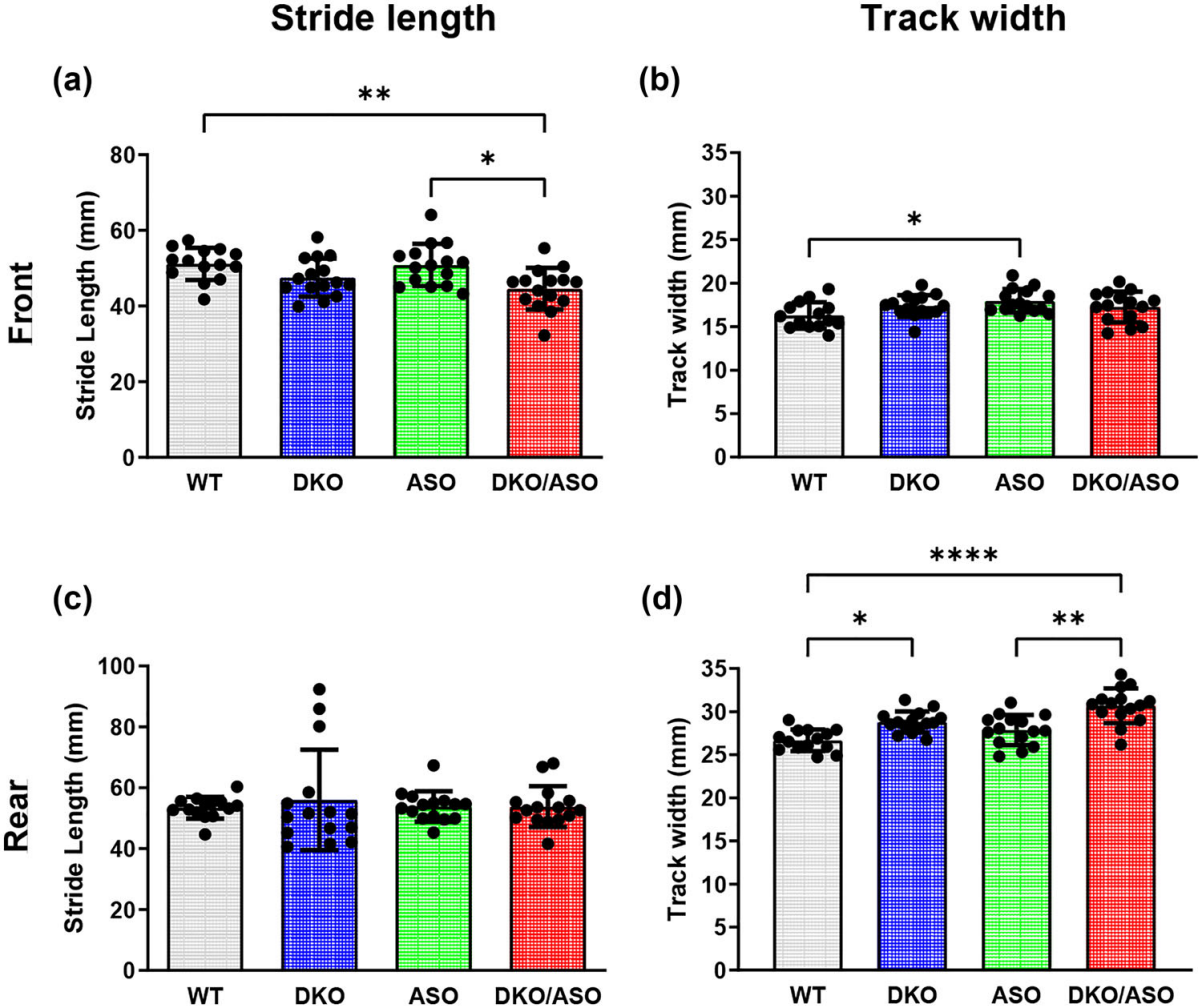
Effect of genotype on gait. (a and c) The average stride length and (b and d) maximal lateral distance for the front and rear limbs were plotted as a measurement of gait. Scatter bars represent the mean ± SD from *n* = 14–15 mice per group. Statistically significant differences are indicated as follows: *p ≤ .05; **p ≤ .01; ***p ≤ .001.

Gait was assessed by measuring both stride length and maximal lateral distance (Figure 8). Although no differences in the hindlimb stride length were detectable, the DKO/ASO group had a significantly shorter forelimb stride length in comparison to the WT and ASO groups (Figure 8a,c; WT, *p* = .0082; ASO, *p* = .0390). Conversely, DKO/ASO mice showed a significantly wider rear track width in comparison to the WT and ASO groups (Figure 8d; WT, *p* < .0001; ASO, *p* = .0042) with no difference in forelimb track width (Figure 8b). DKO mice also displayed a significantly wider rear track width in comparison to the WT group (Figure 8d; WT, *p* = .0202). ASO mice showed no significant changes in rear track width. This differs from the significantly wider front track width in comparison to WT mice (Figure 8b; WT, *p* = .0455).

### 3.9 Effect of genotype on the expression of phosphorylated αSyn

**FIGURE 9:**
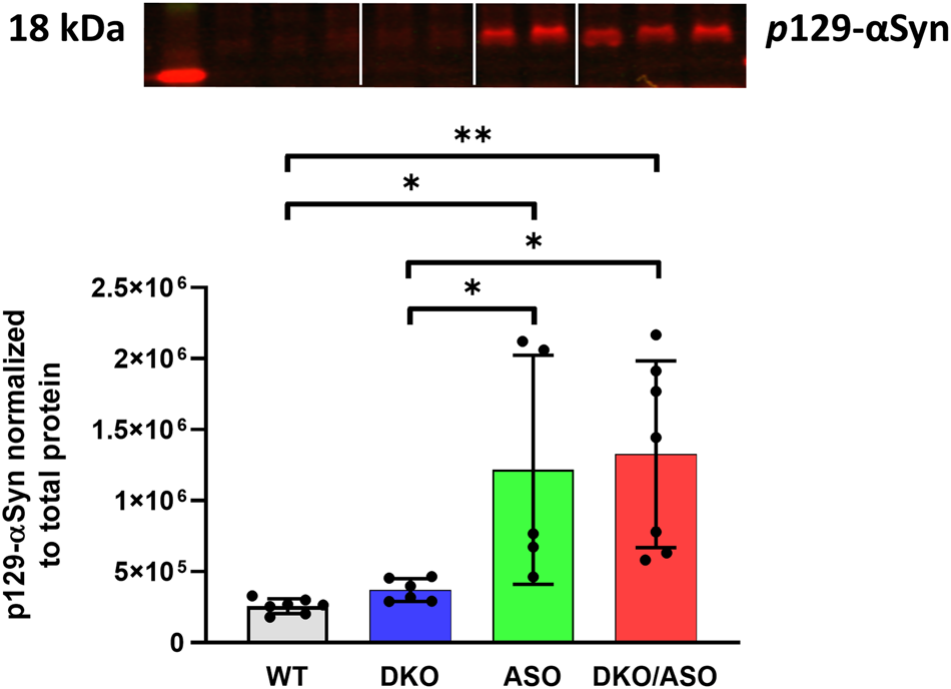
Effect of genotype on midbrain phosphorylated alpha‐synuclein (αSyn). pS129‐αSyn was measured in midbrain tissue from DKO, ASO, DKO/ASO, and WT controls by SDS‐PAGE/western blot (ab51253, 1:2500). The representative bands detected at 18 kDa were visualized using the LiCor Odyssey system and then quantitated. Scatter bars represent the mean ± SD from five to seven samples per group. Statistically significant differences are indicated as follows: *p ≤ .05; **p ≤ .01.

Semi‐quantitative protein expression of phosphorylated αSyn in the striatum is shown in Figure 9. Phosphorylated αSyn at position 129 (pS129‐αSyn) was measured by SDS‐PAGE/western blot in DKO, ASO, DKO/ASO, and WT controls at 6 months of age. The ASO group had a significantly increased accumulation of pS129‐αSyn in comparison to WT (*p* = .017) and DKO (*p* = .0049) groups, but no difference in comparison to the DKO/ASO group (*p* = .981). A significant accumulation of pS129‐αSyn was observed in the DKO/ASO group in comparison to WT (*p* = .0003) and DKO (*p* = .012) groups.

### 3.10 Effect of genotype on the expression of tyrosine hydroxylase

**FIGURE 10:**
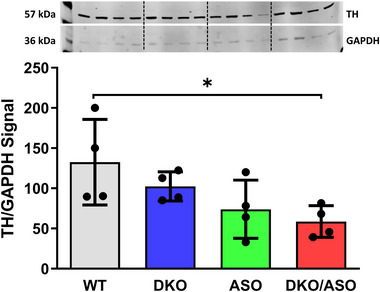
Effect of genotype on the protein expression of striatal tyrosine hydroxylase. Semiquantitative western blot analysis of (a) tyrosine hydroxylase protein expression in the striatum of WT, DKO, ASO, and DKO/ASO mice. GAPDH was used as a loading control. The bands represent tyrosine hydroxylase and GAPDH detected at 57 kDa and 36 kDa, respectively. Scatter bars represent the mean ± SD from *n* = 4 per group. Asterisks represent significant differences as follows: *p ≤ .05.

The effect of genotype on striatal tyrosine hydroxylase (TH) protein expression is shown in Figure 10. A significant effect of genotype on TH expression (*F*_3,12_ = 3.499, *p* = .0497) was observed. Post hoc analysis showed a significant reduction in TH protein expression in the DKO/ASO group in comparison to the WT group (*F*_3,12_ = 3.499, *p* = .0476).

### 3.11 Effect of genotype on the expression of GFAP

**FIGURE 11:**
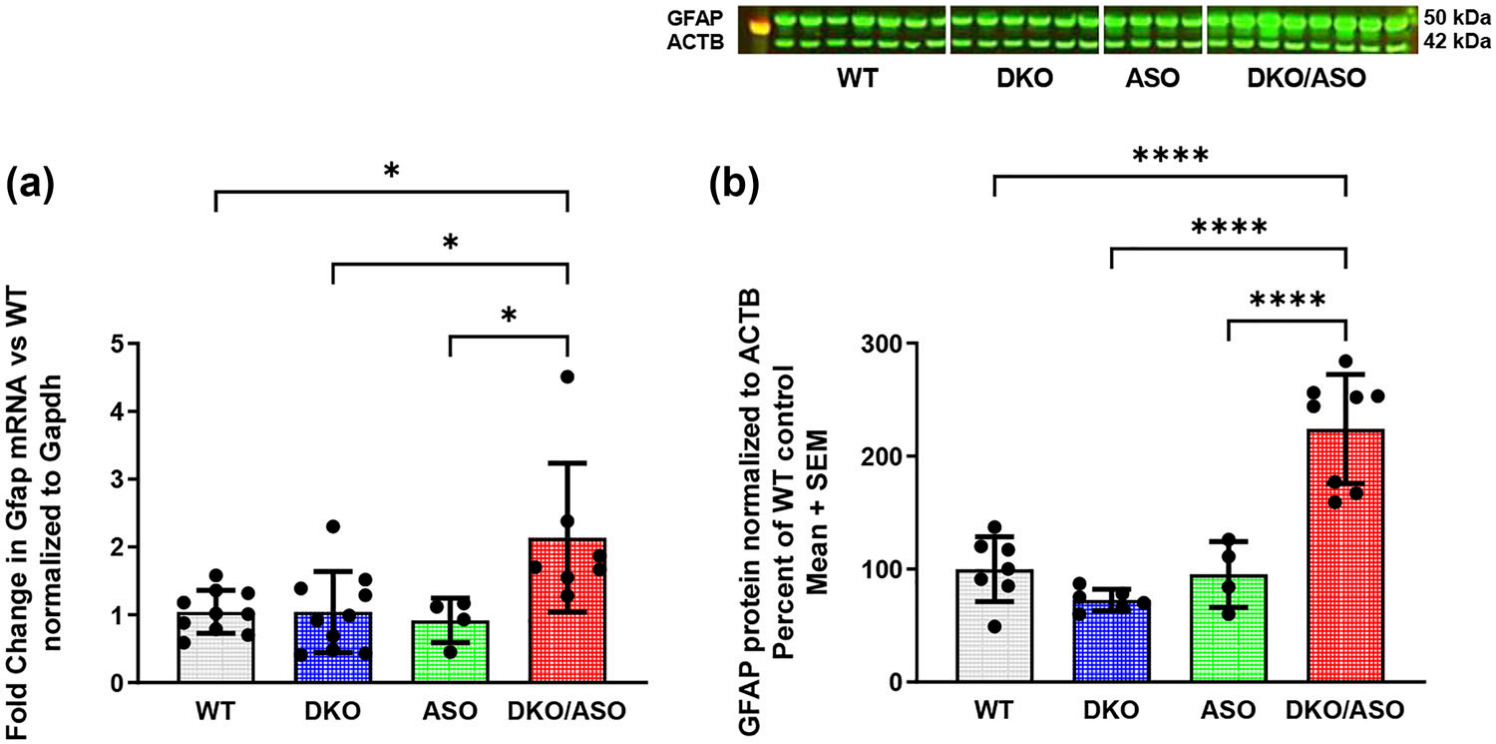
Effect of genotype on expression of striatal Glial fibrillary acidic protein ( GFAP). Fold change of GFAP mRNA expression relative to WT mice was analyzed by quantitative PCR (qPCR) (a), and the semi‐quantitation of GFAP protein expression (b) was analyzed by western blot in the striatum of WT, DKO, ASO, and DKO/ASO mice. The bands represent GFAP and ACTB detected at 50 kDa and 42 kDa, respectively. GAPDH and actin were used as loading controls for qPCR and western blot, respectively. Scatter bars represent the mean ± SD from *n* = 4–10 per group. Asterisks represent significant differences as follows: *p ≤ .05; ****p ≤ .0001.

Figure 11 shows measurements of striatal GFAP mRNA and protein expression. As shown in Figure 11a, the DKO/ASO group had significantly greater fold increase in GFAP mRNA in comparison to the WT (*p* = .0342), DKO (*p* = .0157), and ASO (*p* = .05) groups. Figure 11b shows a similar pattern for protein expression in DKO/ASO mice with significantly greater GFAP expression compared to the WT (*p* < .0001), DKO (*p* < .0001), and ASO (*p* < .0001) groups.

### 3.12 Effect of genotype on locomotor activity and body weight

Locomotor activity and body weight are possible confounding factors in motor performance in our behavioral paradigm. A significant effect of genotype on spontaneous ambulation (*F*_3,55_ = 22.76, *p* < .0001) and rearing (*F*_3,55_ = 24.32, *p* < .0001) was observed. Figure S1a shows ambulatory activity, and Figure S1b shows rearing activity as measured by 24‐h spontaneous locomotor activity. Compared to WT controls, DKO mice display decreased ambulation at 1, 2, and 3 h (*p* < .0001, *p* < .0001, and *p* = .003) and rearing at 2 and 3 h (*p* < .001, *p* = .0064). The ASO group showed increased ambulatory activity at 1, 2, 3, 4, 9, 10, 11, 12, 14, and 15 h (*p* < .0001, *p* < .0001, *p* < .0001, *p* = .0002, *p* = .0013, *p* < .0001, *p* < .0001, *p* < .0001, *p* = .0281, and *p* = .0182) and rearing at 2, 3, 4, 9, 10, 11, and 12 h (*p* = .0096, *p* = .0002, *p* = .0016, *p* = .0097, *p* = .0083, *p* < .0001, and *p =*.0002). Similarly, the DKO/ASO group displayed increased ambulation at 9, 11, 12, 13, and 14 h (*p* = .0055, *p* < .0001 *p* = .0002, *p* = .0094, and *p =*.0151) and rearing at 1, 4, 8, 9, 10, 11, 12, 13, 14, 15, 16, 22, and 23 h (*p* = .0032, *p* = .0146, *p* = .0225, *p* < .0001, *p* = .0054, *p* < .0001, *p* < .0001, *p* < .0001, *p* = .0003, *p =*.0442, *p* = .0131, *p* = .0399, and *p* = .0121).

Figure S2 shows the effect of genotype on body weight. An effect of genotype on body weight was detected (*F*_3,55_ = 8.003, *p =*.0002). Post hoc analysis showed a decrease in body weight in the DKO, ASO, and DKO/ASO groups (*p* = .0006, *p =*.0218, and *p* = .0003).

## 4 DISCUSSION

αSyn variants designed from site‐directed mutagenesis or structure‐based design of both monomeric and fibrillar forms have been utilized to investigate highly unstable and transient conformations of αSyn. αSyn variants that strongly formed oligomers blocked β‐sheet formation observed in mature fibrils and produced toxicity in vitro and when injected in the substantia nigra of rats. In contrast, αSyn variants that formed fibrils were less toxic (Karpinar et al., 2009; Winner et al., 2011). Pathological conditions, such as oxidative stress, may drive the aggregation process and the formation of oligomeric species of αSyn. Evidence from in vitro studies suggests that DOPAL, which accumulates in the postmortem PD brain (Mattammal et al., 1993), is capable of adducting and subsequently cross‐linking αSyn resulting in stable oligomeric conformations (Burke et al., 2008; Doorn & Petersen, 2002; Anderson et al., 2011; Jinsmaa et al., 2011; Nasstrom et al., 2011; Qin et al., 2007; Rees et al., 2009; Werner‐Allen et al., 2016). DOPAL‐induced αSyn oligomers were not only toxic, but they also inhibit the formation of mature fibrils (Burke et al., 2008; Follmer et al., 2015). Moreover, DOPAL modification to monomeric and oligomeric forms of αSyn alters αSyn function in vitro. Together, these data suggest that DOPAL adducts and oligomeric forms of αSyn are the more toxic species. However, the mechanism of neurotoxicity in vivo is unclear. In the present study, our aim was to develop a new mouse model to test the hypothesis that DOPAL enhances αSyn oligomerization to exacerbate motor impairment in mice that overexpress human WT αSyn. To that end, we cross‐bred ASO mice with DKO mice to generate DKO/ASO mice.

We found elevated levels of DOPAL in both DKO and DKO/ASO mice that were deficient in aldehyde degrading enzymes, Aldh1a1 and Aldh2. These results are in line with our previous study indicating that increased levels of DOPAL contribute to pronounced motor deficits in 12‐ to 14‐month‐old DKO mice. In the current study, DKO mice show altered gait and a significant reduction in ambulation and rearing as observed in locomotor activity. Despite these changes, there were no signs of motor impairment on more complex motor tasks. These minor motor disturbances in DKO mice this early in life are in line with our previous report (Wey et al., 2012).

Overexpression of hWT‐αSyn in ASO mice has been reported to result in impaired motor performance on the rotarod as early as 2 months old (Rabl et al., 2017), delayed turn time on the pole test, and adhesive removal time in the adhesive removal test (Fleming et al., 2004). We found that ASO mice display impaired behavior on the pole test and adhesive removal test at 4 and 6 months, respectively. In contrast to results reported by Rabl et al. (2017), deficits in motor‐related behavior were not detected on the rotarod. Over the course of a 5‐day training period, we found that all genotypes improve on the rotarod test. Impaired behavior observed in the ASO group on day 1 was lost by day 3 and remained the same through test day (data not shown). The results represented here rule out any differences in motor performance that may have been confounded by learning over the 5‐day training period.

Impaired behavior of ASO mice on the pole test arise from the lower turn percentage that reflects increased falls in this group. However, despite the reduced turn percentage, ASO mice show no shortcoming in the ability to turn. The turn time may be the more critical component of the test due to the complexity of the task. In contradiction to the results herein, Fleming et al. (2004) reported a delay in turn time on the pole test in 3‐ to 4‐month‐old ASO mice. Differences in behavioral paradigms and/or analyses may explain these contrasting results. In the adhesive removal test, the ASO group showed significant impaired behavior, taking longer to complete the task. This impaired behavior persisted after correcting for contact time. These results are in line with the previous report by Fleming et al. (2004).

DKO/ASO mice were generated to examine the effect of DOPAL on motor performance in mice overexpressing hWT‐αSyn. These mice are observed to have significantly greater motor impairment in comparison to DKO and ASO mice that are solely deficient in aldehyde detoxifying enzymes or overexpress hWT‐αSyn, respectively. Where impaired motor behavior was absent in all other groups, the DKO/ASO group spent significantly less time on the rod than any other group. Similar to the ASO group, a lower percentage of DKO/ASO mice completed the turn component of the pole test. Both groups overexpressing αSyn resulted in more falls and less turns in the pole test. The latency to turn from completed turns indicate that the DKO/ASO group, but not ASO group, take significantly longer to turn in this component of the task. Together these data may suggest that the latency to turn is the more critical component of the pole test. This is backed by the results of the adhesive removal test. The adhesive removal test is a sensorimotor test that may be more sensitive to more subtle disturbances in the nigrostriatal pathway (Fleming et al., 2013, 2004; Lu et al., 2009). Orofacial and forelimb movement required for the adhesive removal test have been linked to dopamine‐dependent behavior (Sager et al., 2010; Sedelis et al., 2000). Sensorimotor tasks include at least two components, detection of the external stimulus through various sensory inputs that begins execution of a motor movement and the complex task of controlling movement to respond to that stimulus (Riemann & Lephart, 2002). Here, results suggest that the detection of sensory stimuli and movement to make contact remains intact in the ASO group, but not in the DKO/ASO group. After correcting for contact time, the latency for both the ASO and DKO/ASO groups to remove the label is significantly increased. This may suggest that the task of removing the adhesive label is more complex and requires greater sensorimotor control.

Disturbances in gait and balance are common in PD (Boonstra et al., 2008; Hausdorff, 2009). PD patients are characterized as having a slow gait that is attributable to a shortened stride length, narrow base, and stooped posture. Reduced foot swing height and stride length lead to a shuffling gait, another feature of PD (Pirker & Katzenschlager, 2017). Similarly, gait disturbances responsive to levodopa treatment have been observed in animal models of PD, including DKO and ASO mice (Amende et al., 2005; Fernagut et al., 2002; Fleming et al., 2004; Wey et al., 2012). Treadmill gait analysis offers automated gait analysis in animals that is translational to clinical studies (Brooks & Dunnett, 2009). In the present study, ASO mice displayed a slight but significant increase in track width in the forelimbs. This contrasts to previously reported data where gait disturbances were absent in 8‐month‐old ASO mice using footprint analysis (Fleming et al., 2004). The DKO group show opposing results to the ASO group with a significant increase in track width in the hindlimbs, but not the forelimbs. Results in the DKO group mirror hindlimb track width differences observed in the DKO/ASO group. This may suggest that elevated aldehydes in the DKO and DKO/ASO groups are driving changes in hindlimb track width. In addition to hindlimb impairment, DKO/ASO mice had a shorter stride length in comparison to WT and ASO mice, but not DKO mice. These observations are partially in line with toxin‐induced gait disturbances, where the hindlimbs are more sensitive to impairment (Fernagut et al., 2002). The broad track width observed in DKO and DKO/ASO groups contrasts to clinical PD where a narrow base is typically observed. The broad base in these groups may be a compensatory mechanism to achieve temporal stability similar to clinical PD where temporal stability is achieved through a higher step frequency and shorter stride length with increasing speed (Morris et al., 1994; Zanardi et al., 2021). It is important to note that while a broad base is uncharacteristic of PD, a broad base is observed in the aged population and individuals with motor impairment (Pirker & Katzenschlager, 2017). Despite minor contrasting results that can be attributed to several factors, these data further suggest that DAergic disruption in the nigrostriatal pathway led to gait disturbances in both the forelimbs and hindlimbs. These disturbances observed in DKO/ASO mice are exacerbated in comparison to mice deficient in aldehyde detoxifying enzymes or mice overexpressing hWT‐αSyn alone.

In the current study, the DKO/ASO group displays significant motor impairment in our behavioral paradigm. Deficits on these motor tasks were not due to impaired learning as we observed in accelerated rotarod training (data not shown), locomotor activity (Figure S1), or body weight (Figure S2), factors known to contribute to poor motor performance (Brooks & Dunnett, 2009; McFadyen et al., 2003). Activity observed in locomotor activity may contribute to altered behavior in other motor tasks. Hyperactivity is observed in both ASO and DKO/ASO groups, while the DKO group showed initial signs of hypoactivity when introduced to a novel environment. Reduced body weight in both the ASO and DKO/ASO groups does not explain poor motor performance on the accelerated rotarod. DKO activity and reduced body weight did not affect other behavioral tasks.

To our knowledge, we are the first to have uniquely detected a deficit accumulation in 4‐month‐old ASO and DKO/ASO mice that parallels deficits in motor performance on the accelerated rotarod, pole test, as well as the adhesive removal test. Frailty increases progressively with increasing age in female C57Bl/6J mice (Whitehead et al., 2014). Frailty index scores for the WT and DKO groups are within range to 5‐month‐old young mice published by Whitehead et al. Nineteen‐month‐old mice were calculated to have a frailty index score of ∼0.11. In this study, 4‐month‐old ASO mice approach this score, while the DKO/ASO group is nearly equivalent to the score observed in 19‐month‐old female mice. With these findings, frailty may provide another means to assess motor‐related behavior in PD without the use of special equipment.

DOPAL, which is hypothesized to accumulate in the PD brain, is known to modify and alter enzymatic activity (Vanle et al., 2017; Vermeer et al., 2012) as well as produce toxicity in vitro and in vivo. This is an effect that has not been observed at physiological concentrations of DA or other DA metabolites (Burke et al., 2008, 2003; Kristal et al., 2001; Panneton et al., 2010). We have previously reported that DKO mice which are deficient in aldehyde detoxifying enzymes accumulate DOPAL in the nigrostriatal pathway as early as 5–8 months old (Wey et al., 2012). This elevation in DOPAL preceded the loss of DAergic neurons and a behavioral impairment in DKO mice. In the current study, heightened DOPAL levels in the DKO group are in line with previous accounts of DOPAL levels in the same line. We, however, did not observe significant motor impairment at an early age. Despite early accumulation of DOPAL, levodopa‐responsive motor deficits become more pronounced at approximately 12 months old (Wey et al., 2012). While motor deficiency in DKO mice is absent at an early age, DKO/ASO mice that are also deficient in aldehyde detoxifying enzymes showed increased DOPAL levels.

It is unclear of the exact mechanism by which DOPAL may be producing its neurotoxic effects and impaired motor‐related behavior. DOPAL has been shown to react with lysine residues leading to protein aggregation (Jinsmaa et al., 2011; Plotegher & Bubacco, 2016; Werner‐Allen et al., 2016). It is noteworthy that αSyn contains 15 lysine residues. In vitro studies have demonstrated DOPAL to adduct and cross‐link αSyn to form toxic oligomers and aggregates (Follmer et al., 2015; Plotegher et al., 2017; Werner‐Allen et al., 2016, 2018). Nigral DOPAL injections in rats resulted in oligomeric species of αSyn and reduced TH immunoreactivity, suggesting the loss of DAergic neurons (Burke et al., 2008). Posttranslational modifications influence the aggregation of proteins (Schaffert & Carter, 2020). Research suggests that serine 129 phosphorylated αSyn (pS129‐αSyn) accumulates in synucleinopathies, including PD (Fujiwara et al., 2002; Halliday et al., 2011). pS129‐αSyn is highly targeted for modification and reported to increase αSyn aggregation in vitro, primarily to form fibrils (Fujiwara et al., 2002). It is reported that pS129‐αSyn accumulates in a progressive manner with soluble and insoluble pS129‐αSyn being significantly greater only in the late stages of PD (Walker et al., 2013). In the current study, the accumulation of pS129‐αSyn in the midbrain of mice overexpressing hWT‐αSyn is in line with a previous report where ASO mice show pS129‐αSyn deposits in the substantia nigra, striatum, cortex, and hippocampus (Chesselet et al., 2012). The undetectable differences in pS129‐αSyn between the ASO and DKO/ASO groups suggest that pS129‐αSyn was not responsible for differential behavior observed in ASO and DKO/ASO groups.

DOPAL may also directly target and alter the enzymatic activity of the DAergic biomarker, TH. Reduced TH expression can result in a decline in DA synthesis that ultimately leads to parkinsonism (Zhu et al., 2012). DKO/ASO mice showed reduced expression of striatal TH as measured by immunoblotting. In previous studies, ASO mice, which develop motor deficiencies as early as 2 months of age, showed no signs of diminished TH or DA until they were 14 months old and no signs of DAergic loss up to 22 months of age (Fleming et al., 2004; Lam et al., 2011). Deficits observed in ASO and DKO groups were exacerbated in DKO/ASO mice.

Chronic neuroinflammation is another possible mechanism involved in the pathophysiology of PD. Increased GFAP expression, which is a biomarker of astrogliosis in neuroinflammation, has been reported in the SN and the striatum of the postmortem PD brain (Lastres‐Becker et al., 2012; Mythri et al., 2011). ASO mice show no indication of increased GFAP levels in the nigrostriatal pathway. However, DKO/ASO mice showed heightened levels of GFAP mRNA and protein. This is similar to what has been reported for 9‐month‐old ASO mice in which there was increased striatal and hippocampal GFAP (Valera et al., 2015). There is some controversy concerning the role that neuroinflammation plays in PD. It is unclear whether the initial protective effects of neuroinflammation exacerbate PD via the overproduction of cytokines and reactive oxygen species (Sanchez‐Guajardo et al., 2013). Thus, further investigation is required to determine whether higher GFAP levels in the DKO/ASO group are a response to injury in the nigrostriatal pathway or if GFAP levels exacerbate PD‐like symptoms.

We recognize that the DKO/ASO mouse model is genetically complex and that no single control group fully explains severe impairment observed in this group. Collectively, however, each group helps us understand the interaction of dopamine aldehyde with alpha‐synuclein. Despite the lack of αSyn brain pathology in the DKO/ASO group, these mice displayed severe motor dysfunction on several frequently used behavioral tests known to be sensitive to DAergic loss that result in impaired motor behavior. Minor behavioral deficits observed in DKO and ASO mice were exacerbated in DKO/ASO mice at 4 and 6 months of age. Greater behavioral deficits in DKO/ASO mice provide evidence consistent with the idea that elevated aldehydes interact with αSyn to exacerbate the behavioral deficits observed in ASO mice, a model known to develop motor symptoms as early as 2 months of age (Chesselet et al., 2012). These results, which may or may not be related to pre‐synaptic DAergic activity, require further investigation. In order to confirm these preliminary findings, further studies are required to determine possible interactions of DOPAL with αSyn in vivo. This would include determining whether neurotoxic αSyn oligomers or neuroinflammation induced by DOPAL is a contributing factor of neurodegeneration and motor impairment.

### PEER REVIEW

The peer review history for this article is available at https://publons.com/publon/10.1002/brb3.3150.

## Supporting information

Figure S1. Effect of genotype on 24‐hour spontaneous locomotor activity.

Figure S2. Effect of genotype on body weight.

## Data Availability

The data that support the findings of this study are available from the corresponding author upon reasonable request.
