# Supplementary figures and images for: Impaired aldehyde detoxification exacerbates motor deficits in an alpha‐synuclein mouse model of Parkinson's disease
Source: Brain Behav. 2023 Jul 14;13(9):e3150. doi: 10.1002/brb3.3150 (PMC10498093; doi:10.1002/brb3.3150)

**a**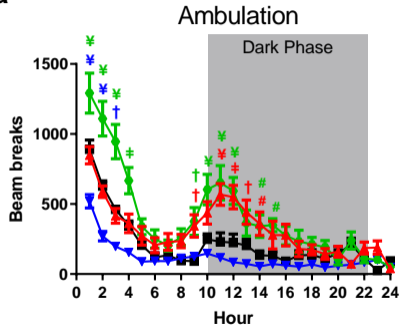**b**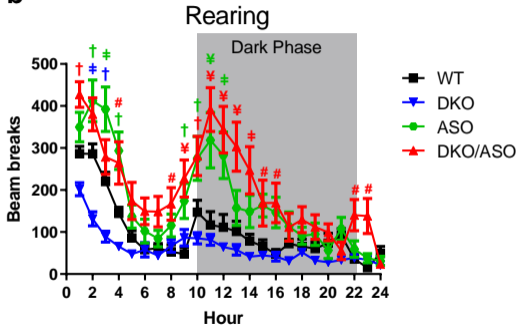

Supplement: Supplementary file 1 — Figure S1. Effect of genotype on 24‐hour spontaneous locomotor activity. [file BRB3-13-e3150-s002.pdf]

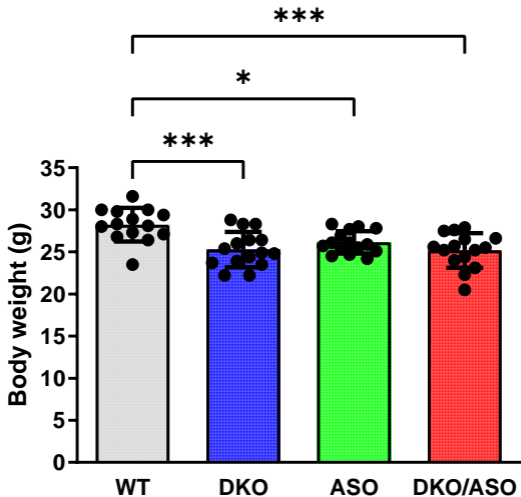

Supplement: Supplementary file 2 — Figure S2. Effect of genotype on body weight. [file BRB3-13-e3150-s001.pdf]
